# A Model in Female Rats With Phenotypic Features Similar to Interstitial Cystitis/Bladder Pain Syndrome

**DOI:** 10.3389/fpain.2021.791045

**Published:** 2021-12-07

**Authors:** Timothy J. Ness, Cary DeWitte, Jennifer J. DeBerry, Morgan P. Hart, Buffie Clodfelder-Miller, Jianguo G. Gu, Jennifer Ling, Alan Randich

**Affiliations:** Department of Anesthesiology and Perioperative Medicine, University of Alabama at Birmingham, Birmingham, AL, United States

**Keywords:** bladder-physiopathology, hypersensitivity, neonatal-methods, interstitial cystitis (IC)/bladder pain syndrome (BPS), visceromotor

## Abstract

This report describes methodological and exploratory investigations of the zymosan-induced neonatal bladder inflammation (NBI) model of interstitial cystitis/bladder pain syndrome (IC/BPS) in female rats. These results validate and extend the currently employed model by evaluating critical timepoints for obtaining treatment effects and identified that a second insult as an adult including repeat intravesical zymosan, intravesical lipopolysaccharide, acute footshock stress, neuropathic nociception (facial) or somatic inflammation (hindpaw) all resulted in magnified visceromotor responses to urinary bladder distension (UBD) in rats which had experienced NBI when compared with their controls. NBI also resulted in increased tone and reactivity of pelvic floor musculature to UBD, as well as increased responsiveness to intravesical potassium chloride solutions, abnormal anxiety measures (elevated plus maze) and an increased number of submucosal petechial hemorrhages following 30 min of hydrodistension of the bladder. These phenotypic findings have correlates to the clinical features of IC/BPS in humans and so support use of this model system to examine mechanisms of and treatments for IC/BPS.

## Introduction

Preclinical models are used to trial potentially effective therapies for painful disorders and to probe mechanisms of these disorders. The best models are those that have the same clinical features as the disorder in humans. A recent scientific panel of the NIH-sponsored multicenter Multidisciplinary Approach to Chronic Pelvic Pain (MAPP) Network (1) identified that non-human animal models of the disorder interstitial cystitis/bladder pain syndrome (IC/BPS) needed to have features of that disorder which included (1) nociception to bladder distension, (2) pelvic nociception and (3) urinary frequency. Since our first report of the neonatal bladder inflammation (NBI) model in 2006 (2) we have been using a “double insult” pretreatment to evoke robust nociceptive responses to urinary bladder distension (UBD). The first insult we utilized consisted of bladder inflammation experienced by rat pups on postnatal days 14-16 (P14-16) which was produced by the intravesical administration of the yeast cell wall component zymosan. The second insult in this model was produced by re-inflammation of the bladder using intravesical zymosan as an adult (age 12–15 weeks) 24 h prior to testing. Using this model with its fixed methodological parameters, we and others have reported that these pretreatments result in an increased vigor of cardiovascular and visceromotor responses (VMRs; abdominal contractions) to UBD, an increase in spontaneous micturition, reduced volume/pressure thresholds for evoking a micturition response in cystometric studies, increased neuropeptide content in the bladder, increased cold sensitivity of the bladder, altered analgesic responses to opioid agonists, altered responsiveness to neuromodulatory manipulations, altered spinal neuronal responses to visceral stimuli and altered GABA-A, KCC2 and VGAT mRNA expression [(3–8); Ness et al., 2021; (2, 9–15)].

In our publications, we have used the term “Neonatal Bladder Inflammation” for this model system following the convention of others [e.g., (16–18)] but recognize that more generic terms such as “Early-in-Life Bladder Inflammation” or “Childhood Bladder Inflammation” may be more appropriate since precise correlates between human and rats in terms of years/days and what constitutes the neonatal period is a matter of debate (19). For purposes of this manuscript, and to be consistent with previous reports, we will generally use the term NBI.

Despite the extensive nature of our previous studies, additional methodological and exploratory questions are still worth addressing. Therefore, the present studies re-examined the zymosan-induced NBI model by performing methodological investigations related to the timing and duration of bladder inflammation required to produce hypersensitivity and determined whether this model was also associated with other stimuli and responses associated with urological and non-urological sensations. To improve clarity of presentation, these studies will be arranged according to individual questions which are then addressed by separate Background, Methods, Results and Summary subsections. This presentation of individual points will then be followed by a more global discussion of the overall model system.

## Question #1: Is P14-16 the Optimal Timepoint for the First Insult?

### Background

Our original studies were designed based on the report by Al-Chaer et al. (18) of their model of irritable bowel syndrome where they administered 2 weeks of colorectal stimulation beginning at postnatal day 8 (P8). It was immediately apparent that our rat pups could not survive more than 3 days of treatment starting at P8 and so the period of treatment was shortened to the middle of our planned time period, on days P14-16. We have reported that similar bladder inflammation treatments administered at P28-30 (2) or P90-92 (4) did not lead to augmented responses as adults. Those studies gave evidence that there exists a critical developmental period where inflammatory events may alter subsequent sensory processing. The present study chose to narrow down the period of time where NBI treatments might be effective at producing augmented responses as adults by comparing effects produced by NBI given on P7-9, P14-16 or P21-23. VMRs to UBD and spontaneous rates of micturition were both examined.

### Methods

#### General

All studies were performed in female Sprague-Dawley rats and were approved by the University of Alabama at Birmingham's Institutional Animal Care and Use Committee. Use of exclusively female rats in this model system was based primarily on the practical issue that cannulation of the urethras of male rats, in our hands, was difficult and often led to local tissue trauma whereas cannulation of the urethras of females was easily performed. There is also the epidemiological justification that painful bladder disorders occur predominantly in females (20) and so, if only one sex were to be studied, it would best be females. To obtain female rat pups, timed pregnant females were obtained from Harlan/Envigo Laboratories (Sprattville, AL) and date of birth verified by daily observation of cages. On a defined postnatal day [P7, P14 or P21] pups were sexed and the male rats culled such that the female rats of these studies were sequestered from subsequent exposure to male rats. As pups, separate groups of the female pups underwent treatments for three consecutive days: P7-9, P14-16 or P21-23. Following each treatment, pups were returned to home cages. Subsequently, rats were raised using standard husbandry methods with weaning from the dams at 3 to 4 weeks of age. All rats were raised to 12-15 weeks of age and then underwent additional testing.

#### Neonatal Treatments

All rats underwent one of two treatments on either days P7-9, P14-16 or P21-23. In the NBI group, rat pups were anesthetized with 2–5% isoflurane in oxygen, injected with ampicillin (50–100 mg/kg s.c.), their urethral orifice swabbed with an iodine-povidone solution and a 24 gauge angiocatheter (or PE10 tubing for P7-9 rats) passed transurethrally into their bladder. A solution of Zymosan A (1% in normal saline; 0.05 ml; Sigma Aldrich, St. Louis, MO) was injected into the bladder and allowed to dwell for 30 min. Pups were kept warm on a heating blanket, allowed to recover and returned to their mothers. Control treatments for NBI (labeled as Anesth-) consisted of a similar anesthetic for 30 min, iodine-povidone swabbing, ampicillin treatments and identical recovery protocols. Cannulation of the urethra and/or administration of intravesical saline was not included in control treatments due to the potential for producing unintended inflammation of the bladder through direct physical interaction. Notably, our initial studies of the NBI model did not observe quantitative differences between intravesical saline-treated rats and those which underwent the control treatments described here (2).

#### Voiding Spot Assay

As adults (12–15 weeks of age) and prior to any other treatments, female rats which had received Neonatal Treatments (preceding section) but no adult treatments were placed in a 20 x 50 cm plastic cage for 6 h beginning at 8:00 AM. The cage had a piece of filter paper [Whatman Filter Paper, Grade 2] covering the flooring. After this session the filter paper was allowed to dry and then examined by an observer blinded to group using ultraviolet light to identify the number of individual urine spots, each indicating a voiding episode.

#### Adult Bladder Inflammation

Similar to the Neonatal Treatments noted above, but as adults (12–15 weeks of age), female rats were anesthetized with 2–5% isoflurane in oxygen, injected with ampicillin (50–100 mg/kg s.c.), their urethral orifice swabbed with an iodine-povidone solution and a 22 gauge angiocatheter passed transurethrally into their bladder. A solution of Zymosan A (1% in normal saline; 0.5 ml; Sigma Aldrich, St. Louis, MO) was injected into the bladder and allowed to dwell for 30 min. Rats were kept warm on a heating blanket, allowed to recover and returned to their home cages. Control adult treatments (labeled -Anesth) consisted of a similar anesthetic for 30 min, iodine-povidone swabbing, ampicillin treatments and identical recovery protocols. ABI/Anesth treatments occurred only once and were performed approximately 24 h prior to VMR testing described below.

#### Visceromotor Response (VMR) Measures

Adult rats were anesthetized with 2–5% isoflurane, and a 22 gauge angiocatheter was placed transurethrally into the bladder and held in place by a tight suture around the distal urethral orifice. Silver wire electrodes were placed in the external oblique musculature immediately superior to the inguinal ligament. Isoflurane was then lowered until flexion reflexes were present (approximately 1% isoflurane). UBDs (20 s, 10–60 mm Hg) were produced using compressed air, and intravesical pressure was monitored using an in-line pressure transducer. Contraction of the abdominal musculature in response to UBD has been well characterized (21–23) and was quantified as electromyographic (EMG) activity measured via the external oblique electrodes using standard differential amplification (Grass, Inc. P511 AC amplifiers; 50 x amplification, 60 Hz clipping, low filter setting 10 Hz–high filter setting 3 KHz). The analog EMG signal was digitized with a sampling rate of 10 KHz and saved on computer with digital rectification via software (Spike 4 software; Cambridge Electronic Design Limited, Cambridge, UK) allowing for calculation of mean EMG activity (in mV) during any defined time period. Responses to UBD were quantified as the mean rectified EMG activity during the 20 s of UBD minus the “basal” mean EMG activity measured during the time period immediately preceding UBD. Notably, for each data set the same amplifier and filter settings were used for all rats. Typically, three repeated 20 s duration UBDs at 60 mm Hg intensity were presented at 3 min intervals and then followed by the measurement of responses to graded constant pressure stimuli (10–60 mmHg, 20 s, 1 min intertrial intervals).

#### Quantitative Analysis

**Figure 1:**
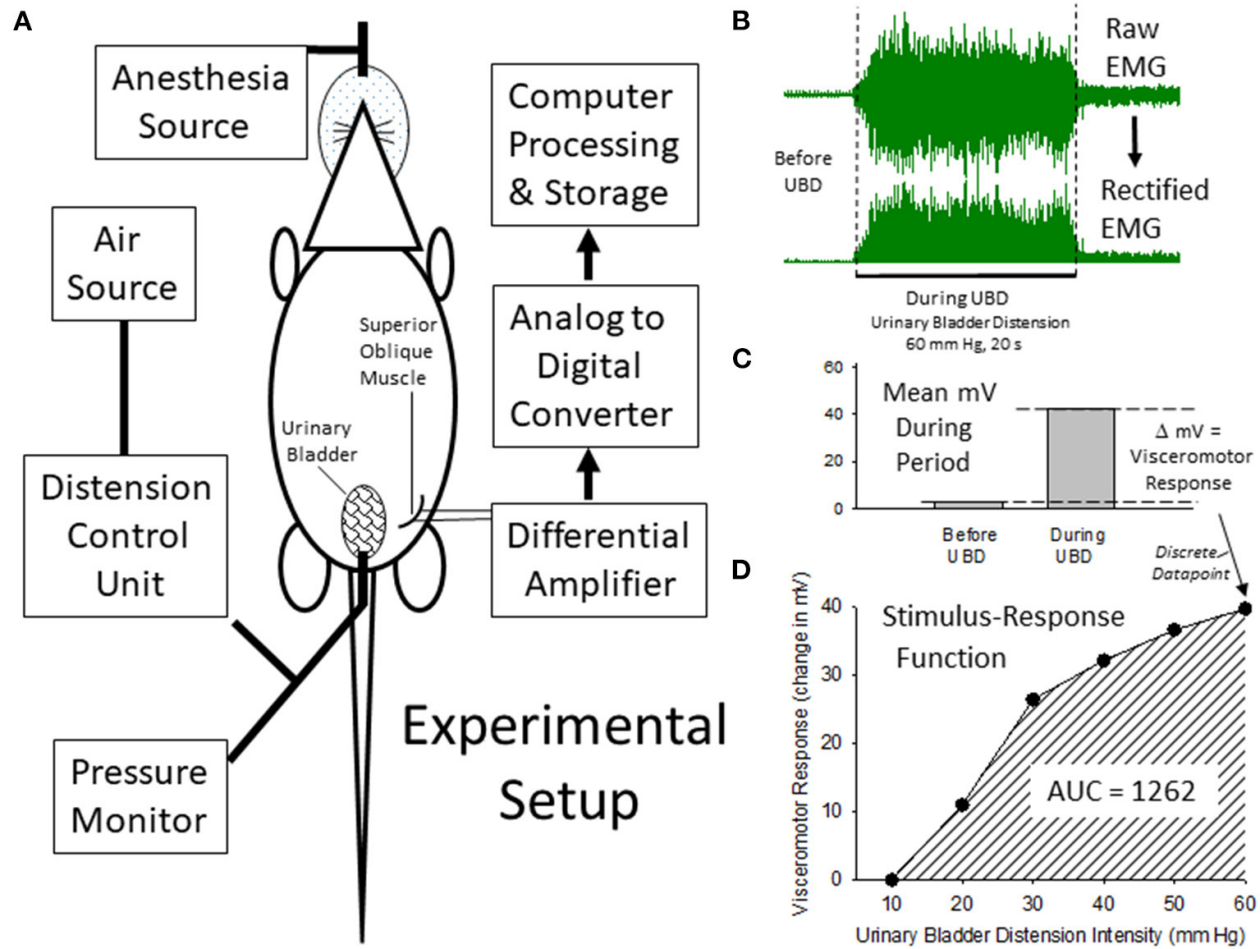
Description of Experimental Setup and Quantitiatve Analysis. **(A)** is a graphical illustration of the equipment and animal resources used for the generation of visceromotor responses to urinary bladder distension (UBD). Air is used to distend the bladder via a transurethral intravesical catheter. Responses are measured as electromyograms (EMG) of the superior oblique abdominal musculature. An example of a digitized EMG before and during UBD at an intensity/duration of 60 mm Hg/20 secs is given in **(B)** as well as its rectified transformation (all negative values made into positive values of equal intensity). These EMG responses were then quantified as the difference between the mean voltages of EMG activity before and during the 20 s of UBD **(C)** to generate a discrete data point that was part of an overall Stimulus-Response Function **(D)** characterizing the individual rat's visceromotor responses to graded UBD. An Area-Under-the-Curve (AUC) statistic related to the Stimulus-Response Function could then be generated which was representative of the overall vigor of the visceromotor responses.

**Figure 2:**
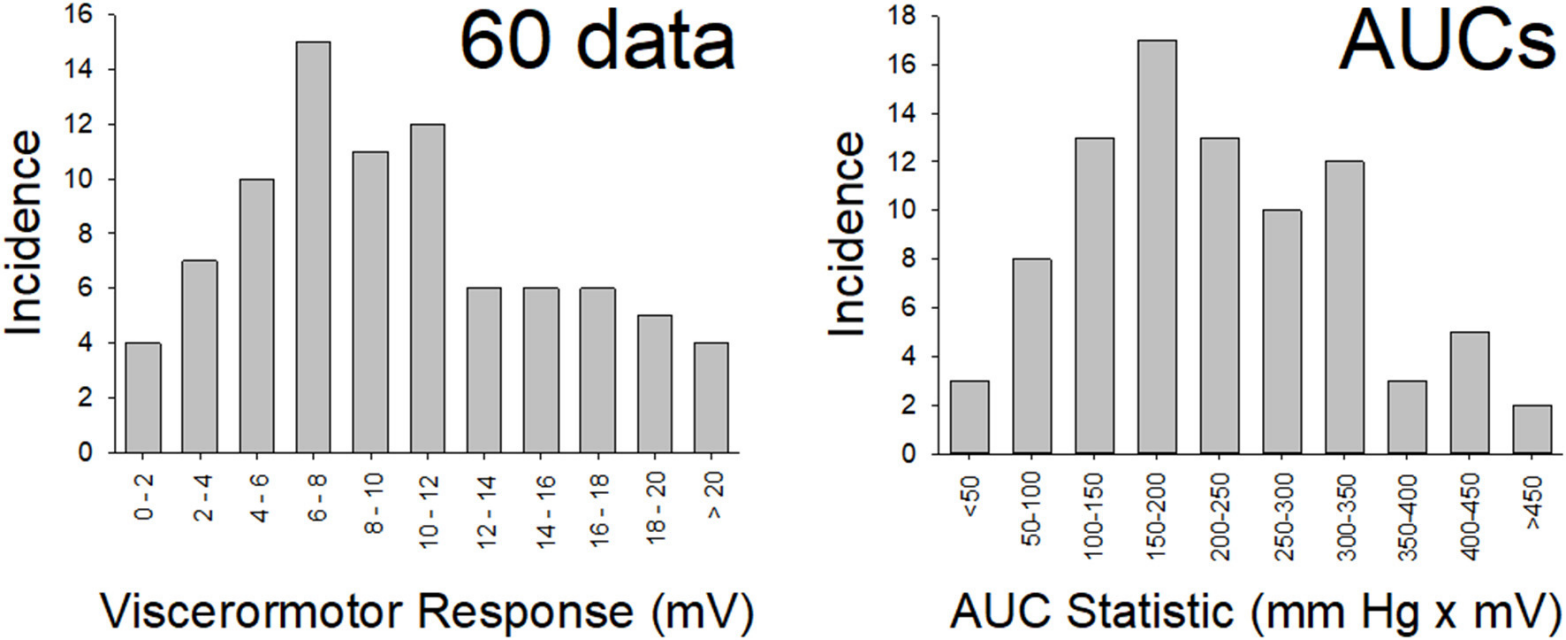
Normal Distribution of Data. Distribution of data from the “control” (*n* = 86) rats in the present study for vigor of visceromotor responses to a 60 mm Hg, 20 s UBD (disrete stiumulus data; left panel) and for the Area-Under-the-Curve measures (overall responses; right panel) to graded UBD. These rats did not experience NBI as pups or any second insult as adults. Both data sets met tests of normality (more explanation and statistics in main text).

In this, as well as subsequent sections of this manuscript, data will be reported as means ± SEM unless otherwise stated. The vigor of VMRs will be expressed as “Visceromotor Response” defined as increases in mean EMG activity during the 20 s period of UBD over the immediate prestimulus level of activity. Logistically, during each experimental run, the differential amplifier output is digitized and rectified by computer software (Spike 2, Cambridge Electronic Design, Inc., Cambridge UK) to positive values, which are then averaged over a period of time to give a mean voltage measure of amplified EMG activity. Measures of evoked activity were then treated as discrete data points and analyzed using a Repeated Measures ANOVA. In addition, when utilizing multiple different intensities of UBD, a stimulus-response function was generated which was then associated with an Area-Under-the-Curve statistic (AUC measure). A demonstration of this analysis, as well as a graphical description of the experimental apparatus related to VMRs is presented in Figure 1. Notably, an analysis of AUCs (generated from individual stimulus-response functions for each animal) as well as each animal's discrete response to a 60 mm Hg, 20 s UBD stimulus were pooled for analysis from the multiple groups of control data in the present study (i.e., rats that only received anesthesia treatments as pups and no active interventions as adults–e.g., saline injection to hindpaw, sham surgery, only anesthesia, etc.). The original generation of these data utilized the same amplifier and processing settings and subsequent analysis demonstrated that these statistics were normally distributed (Figure 2). Specifically, the data associated with the discrete VMRs to a 60 mm Hg, 20 UBD generated a Shapiro-Francia statistic of W' = 0.9527, an Anderson-Darling statistic of W = 1.2049 and a Kolmogorov-Smirnov Test statistic D = 0.11341. Similarly, the data of AUC measures for these same rats was the following: Shapiro-Francia W' = 0.9138; Anderson-Darling W = 1.7073; Kolmogorov-Smirnov D = 0.10303. These statistics all justify use of parametric statistical analyses. Notably, use of the AUC statistics allowed for an easily interpretable visualization of the overall effect of the interventions (e.g., 4 bar graphs for 2 x 2 conditions of NBI/ABI as opposed to 24 separate data points for 6 intensities of UBD in a 2 x 2 condition) while generating the same probability statistic as a more complex analysis (e.g., a one-way ANOVA comparison of the AUCs for different conditions generates a probability estimate identical to that of an overall repeated measures ANOVA comparing data related to multiple conditions and multiple intensities of UBD. For this reason, the statistics associated with the simpler AUC data analysis are presented unless stated otherwise.

### Results

#### Voiding Episodes Are More Numerous in Rats Treated With Zymosan on P14-16 and P21-23

Data described in Table 1 (number of subjects for each condition/timepoint, number of voids) indicate the Total Number of Voids measured in adult female rats treated with intravesical zymosan on either days P14-16 or P21-23 was statistically greater than the Total Number of Voids in their corresponding neonatal Anesth- control groups which did not experience bladder inflammation (*p* = 0.003 for P14-16 groups; *p* = 0.015 for P21-23 groups; unpaired *t*-test comparisons). Rats experiencing zymosan-induced NBI on days P7–9 were not statistically different than their corresponding neonatal Anesth- control rats (*p* = 0.275; unpaired *t*-test comparison). A more sophisticated analysis of void spots (e.g., size of each spot which correlates with volume of void) was not performed but could be utilized in future studies.

**Table 1:** Voiding spot assay measures in adults female rats which received Early-In-Life (EIL) bladder treatments.

**Treatment Group**	***n***	**Number of Voids**
P7-9 Zymosan	32	10.8 ± 1.2
P14-16 Zymosan	16	19.4 ± 2.0^**^
P21-23 Zymosan	14	17.9 ± 2.5^*^
P7-9 Control	25	12.7 ± 1.2
P14-16 Control	16	10.1 ± 1.1
P21-23 Control	14	10.3 ± 1.5

*Data represents mean ± SEM*.

* and ** *indicate significant difference from corresponding control group (p < 0.05 and p < 0.01, respectively; unpaired t-test)*.

#### VMRs More Robust in Rats Treated With Zymosan on P14-16

**Figure 3:**
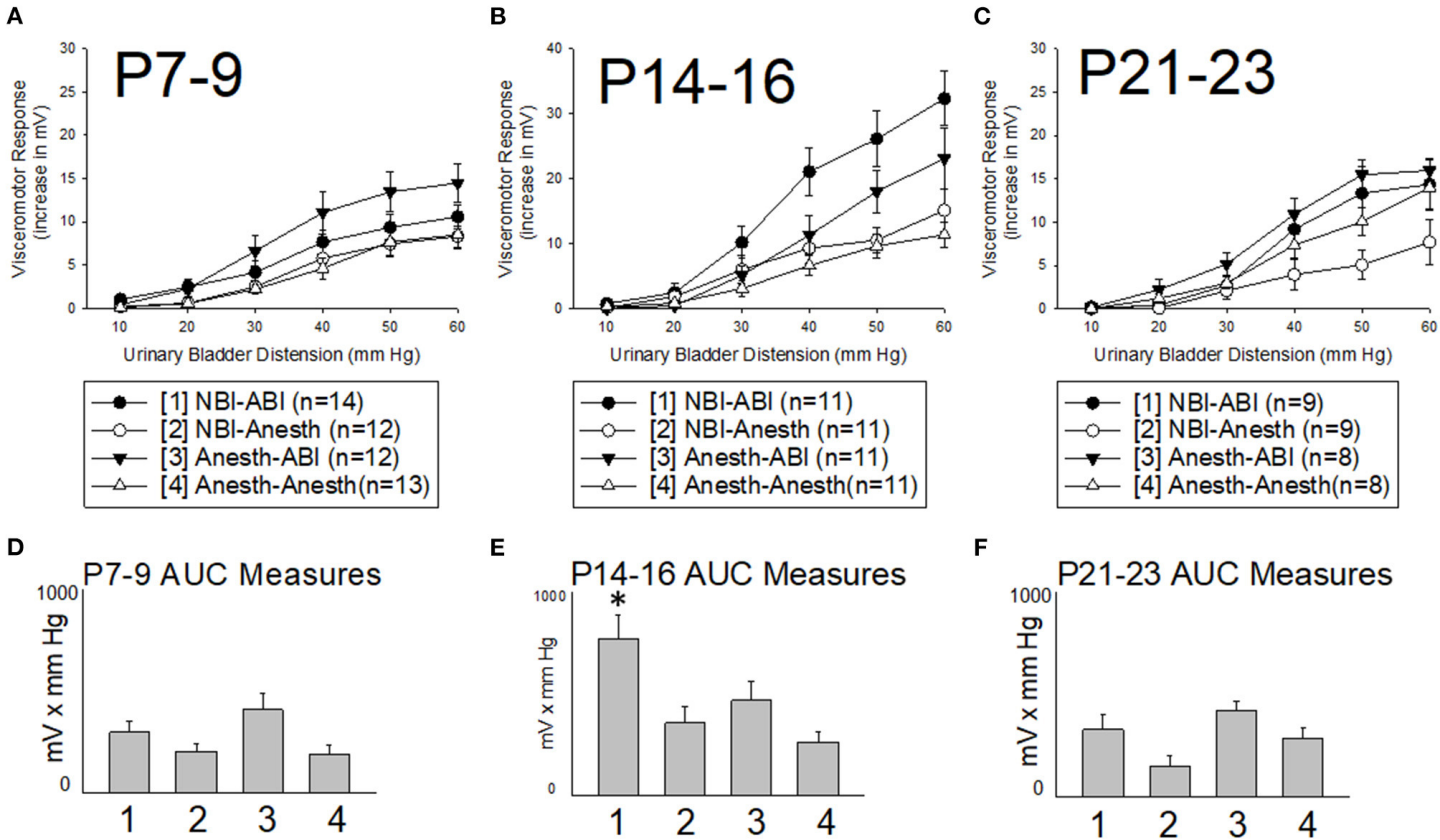
Effect of Timing of Neonatal Treatments on Visceromotor Responses as Adults. All panels are labeled by their 3 days of postnatal treatment as pups [P7-9, **(A,D)**; P14-16, **(B,E)**; P21-23, **(C,F)**] with either intravesical zymosan producing neonatal bladder inflammation (NBI treatment) or with control treatments (Anesth treatment). One day prior to study, adults rats received either retreatment with intravescial zymosan producing acute adult bladder inflammation (-ABI treatment) or with control treatments (-Anesth treatment). All rats received a combination of an adult and a neonatal treatment as labeled. Y-axis in **(A–C)** indicate the vigor of the visceromotor responses measured as increases in abdominal electromyographic activity evoked by the intensities of urinary bladder distension indicated in the x-axis. **(D–F)** indicate Area-Under-the-Curve (AUC) measures for the stimululs-response functions in the main panels–pretreatment groups are indicated numerically as per the legend box. Although ABI produced statistically significant increases in visceromotor activity with treatment at all three timepoints, only treatment at the P14-16 timepoint led to an increase in responses of adult rats in the NBI-ABI group when compared with the Anesth-ABI group. Data is presented as means ± SEM. N's and treatment groups are as indicated in legend boxes. * in **(E)** indicates statistically significant difference from the other three groups with *p* < 0.05.

VMRs were evoked by UBD at intensities ranging from 10–60 mm Hg leading to stimulus-dependent increases in abdominal EMG activity (Figure 3). These reflexes were most robust in rats which had been treated on days P14-16 with intravesical zymosan (neonatal bladder inflammation; NBI) and then retreated as adults with intravesical zymosan (adult bladder inflammation; ABI). A comparison of all rats treated on days P14-16 which subsequently received ABI as an adult (combination of NBI-ABI and Anesth-ABI groups) with their appropriate control groups (NBI-Anesth and Anesth-Anesth groups) demonstrated a statistically significant effect of ABI (*p* = 0.021) supporting previous observations that ABI as an acute insult produces hypersensitivity (2, 24). More importantly, a comparison of all rats treated on P14-16 which received NBI as a neonate (combination of NBI-ABI and NBI-Anesth groups) with their appropriate control groups (Anesth-ABI and Anesth-Anesth groups) also demonstrated a statistically significant effect of NBI (*p* = 0.048). As is apparent in Figures 3B,E, this effect was mostly due to the NBI-ABI subgroup. *Post hoc* analysis of the data from rats treated on days P14-16 demonstrated the NBI-ABI group differed significantly from the Anesth-ABI group (difference *p* = 0.044), the NBI-Anesth group (*p* = 0.009) and Anesth-Anesth group (*p* = 0.001). A similar examination of data from the P7-9 (Figures 3A,D) and P21-23 groups (Figures 3C,F) did not demonstrate any *increases* in vigor of VMRs due to NBI treatment but did demonstrate effects of ABI (P7-9 data *p* = 0.010; P21-23 data *p* = 0.012). Interestingly, the P21-23 group had a small, but statistically significant decrease in evoked VMR activity as an effect of NBI (*p* = 0.026) when doing the same analysis.

### Interval Summary

Of the three timepoints studied, the only neonatal treatment period in which NBI resulted in both increases in spontaneous micturition and increased robustness of VMRs to UBD as adults was the P14-16 timepoint. This supports methodological use of the P14-16 timepoint and narrows the “window of susceptibility” for the critical period of development associated with the NBI effect from <28 days to the time period of >9 to <21 days.

## Question #2: Are Three Days of Intravesical Zymosan Treatment Needed?

### Background

As noted earlier, we settled on a 3 day Neonatal Treatment paradigm by empiric determinations related to subject survivability. This treatment is time consuming and means that the pups are exposed to multiple antibiotic doses, multiple instrumentations of their urethras and multiple episodes of anesthesia. It was possible that a single bout of NBI would be sufficient to observe the multiple effects that we had noted with 3 days of treatment and so a brief set of experiments was performed to determine whether 1 day of treatment might be sufficient.

### Methods

Methods identical to those described in section Neonatal Treatments were utilized with the following modifications: (1) only intravesical zymosan treatments (NBI) beginning on P14 were employed and (2) the female rat pups treated with NBI were separated into two groups, one of which received a single day of NBI on P14 and the other group which received 3 days of NBI on P14-16 identical to the above described protocols. ABI and VMR protocols identical to those described in Sections Adult bladder inflammation and Visceromotor Response (VMR) Measures were also employed and statistical analysis performed as in Section Quantitative analysis.

### Results

**Figure 4:**
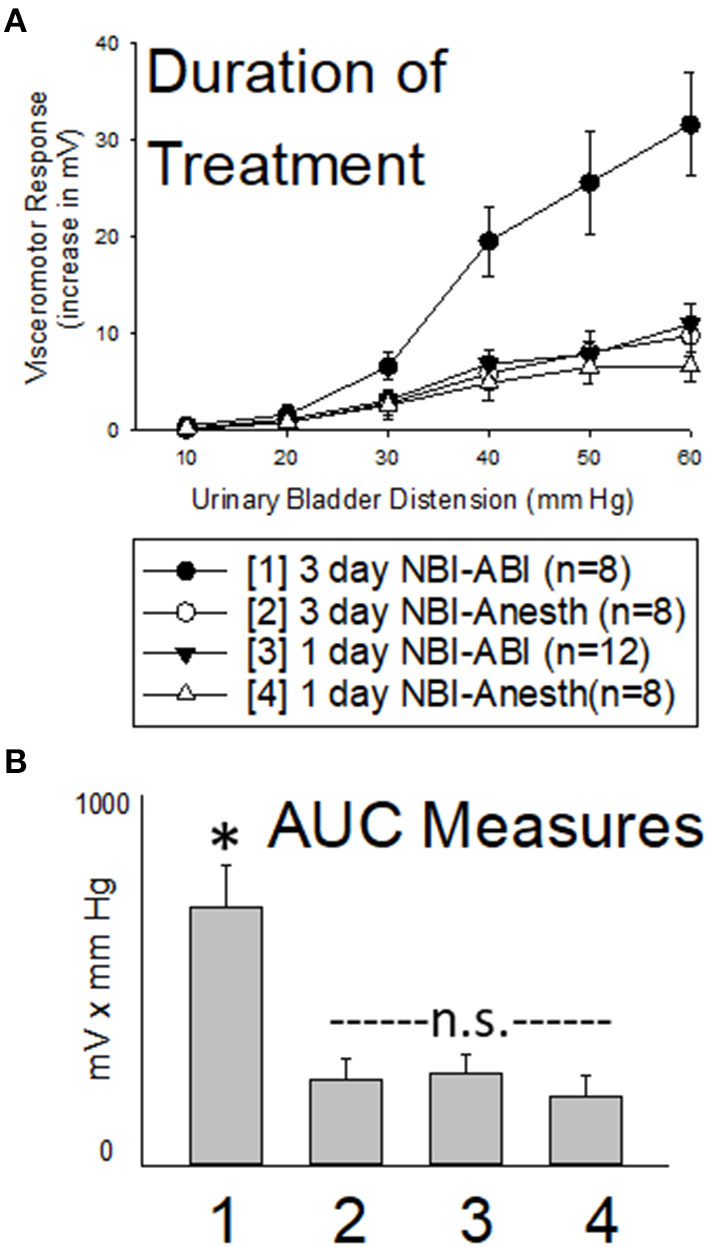
Effect of Altering Duration of Neonatal Bladder Inflammation (NBI) Treatment. Data presented in same fashion as panels in Figure 2 (group stimulus-response functions in **(A)**; Area-Under-the-Curve, AUC, measures in **(B)**). Three days of NBI treatment followed by adult bladder inflammation (ABI) resulted in statistically more robust visceromotor responses when compared with all other groups tested. Stated statistics represent ANOVA results. * indicates 3 day NBI group different from all other groups (*p* < 0.05) for data point indicated; “n.s.” indicates non-significant difference from other groups indicated.

As is apparent in Figure 4, one day of NBI treatment coupled with either ABI or an adult control treatment (the 1 day NBI-ABI and 1 day NBI-Anesth groups) did not result in an apparent augmentation of VMRs. However, 3 days of NBI treatment coupled with ABI (the 3 day NBI-ABI group) produced VMRs that were statistically more robust that the other three groups studied (comparison with 1 day NBI-ABI group *p* = 0.006, with 3 day NBI-Anesth group *p* = 0.005 and with 1 day NBI-Anesth group *p* = 0.003). However, data from those other groups were not statistically different from each other.

### Section Summary

A single 30 min treatment on P14 with intravesical zymosan did not produce robust increases in VMRs to UBD in adult rats whereas three repeated daily treatments on P14-16 did produce more robust VMRs than their controls. This supports use of a 3 day NBI treatment protocol.

## Question #3: Can Bacterial Lipopolysaccharide Also Act as an Appropriate First Insult?

### Background

Our previously reported studies noted in the Introduction section used zymosan to induce bladder inflammation. Derived from yeast cell walls, zymosan is an activator of TLR2 and TLR6 receptors (25) and produces a robust local inflammatory effect in the bladder (24). We had previous experience with its use in somatic systems (26, 27) and it was used by others as an inflammatory agent of the colon/rectum [e.g., (28)]. Bacterial lipopolysaccharide (LPS) derived from *E.Coli* also seemed like an appropriate substance to study the effects of NBI since bladder infections are common in childhood, especially in females (29) and *E.Coli* is a common source of urinary tract infections (30). LPS is an activator of TLR4 receptors (25) and we chose a specific *E.Coli* strain-derived type of LPS that has been well characterized and utilized by multiple laboratories to induce robust bladder inflammation in rodents [e.g., (31)]. Our overall investigative plan was to compare and contrast use of LPS with zymosan as intravesical agents in our NBI model.

### Methods

Methods identical to those described in section Neonatal Treatments were utilized with the following modifications: (1) only treatments on P14-16 were employed and (2) the female rat pups treated with NBI were separated into two groups, one of which received zymosan (as before; in these studies designated as ZY.NBI) as the intravesical agent and the other group which received LPS (0.05 ml, 30 min instillation, 100 μg/ml, *E.Coli* strain source O55:B5, Sigma-Aldrich, St. Louis, MO; in these studies; designated as LPS.NBI). Neonatal control groups (Anesth) were treated as before. ABI protocols identical to those described in Sections Adult bladder inflammation were also employed with the modification that some rats were treated with intravesical zymosan (as before; designated + ZY.ABI) and others were given a single dose of intravesical LPS (0.5 ml, 30 min instillation, same source/strain as in the NBI treatments; designated + LPS.ABI) approximately 24 h prior to testing. Adult control groups (+ Anesth) were treated as before. VMR protocols identical to those described in section Visceromotor Response (VMR) Measures were employed and statistical analysis performed as in section Quantitative analysis.

### Results

**Figure 5:**
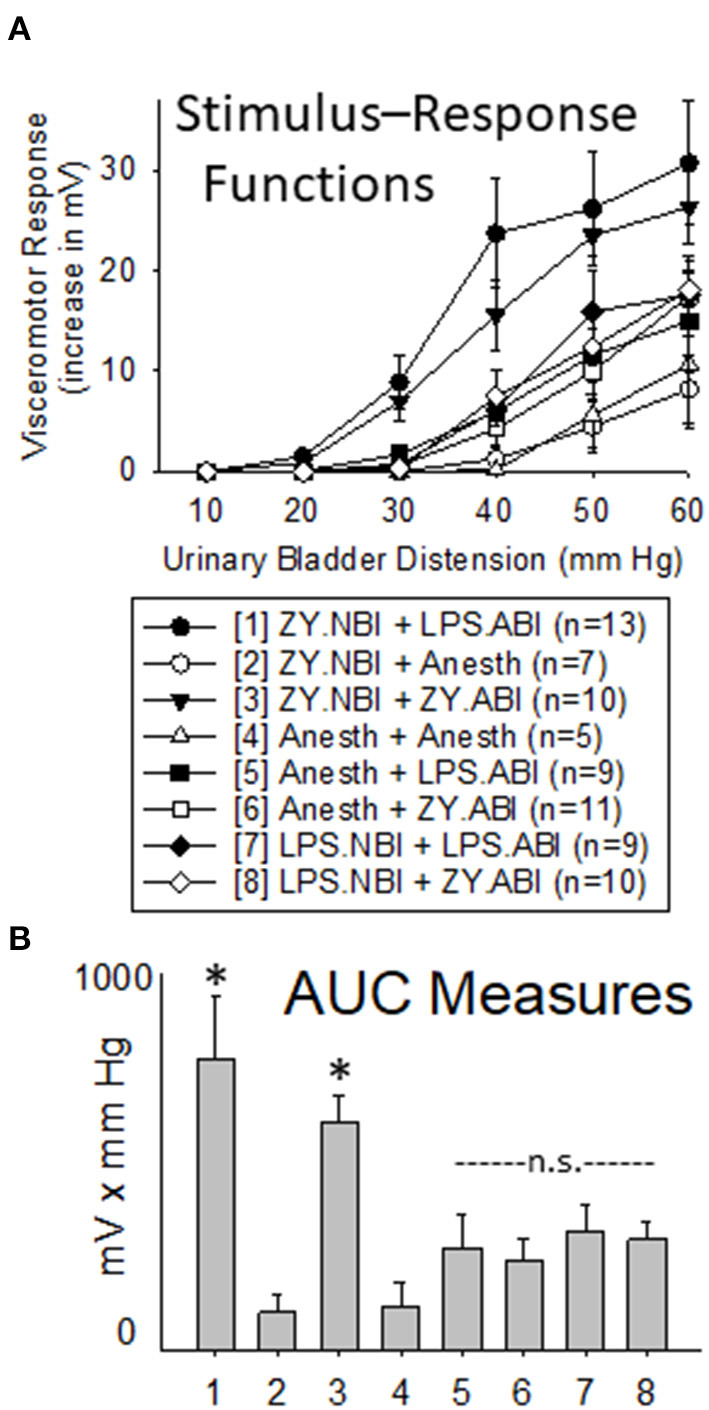
Comparison of Use of Intravesical LPS vs. Zymosan for NBI on VMRs to UBD in Adult Rats. Presentation of data is similar to that in Figures 2, 3 (group stimulus-response functions in **(A)**; Area-Under-the-Curve, AUC, measures in **(B)**). “ZY.NBI” and “LPS.NBI” indicate animals in those groups experienced NBI induced by intravesical treatments with zymosan and LPS respectively. Similarly, “ZY.ABI” and “LPS.ABI” similarly indicate adult intravesical treatments producing ABI. “Anesth” indicates control treatments at neonatal and adult timepoints. The “ZY.NBI + LPS.ABI” (Group 1) and “ZY.NBI + ZY.ABI” (Group 3) data were statistically more robust than responses from each of the other groups, but not from each other (Repeated Measures ANOVA; * indicates *p* < 0.05 for comparison with all other groups). Notably, the robustness of visceromotor responses of the “Anesth + ZY.ABI”, “Anesth + LPS.ABI”, “LPS.NBI + ZY.ABI” and “LPS.NBI + LPS.ABI” (Groups 5–8) did not statistically differ from each other and are indicated as “n.s” for non-significant.

Intravesical LPS administration to adult rats (+ LPS.ABI groups) produced an augmentation of VMRs when compared with adult controls (+ Anesth groups) indicating that the dose and route of LPS was capable of producing effects. Notably, this augmenting effect of LPS was most robust in rats which had received NBI using zymosan (the “ZY.NBI + LPS.ABI group” when compared with “ZY.NBI + Anesth” group was different with *p* = 0.011), an effect which was comparable in magnitude to the augmentation produced by zymosan-induced ABI in rats which had received NBI using zymosan (the “ZY.NBI + ZY.ABI group” compared with “ZY.NBI + Anesth group” was statistically different with *p* = 0.001). However, it is notable that rats which received LPS for induction of NBI (the LPS.NBI + LPS.ABI and LPS.NBI + ZY.ABI groups) did not demonstrate augmented responses following ABI when compared with rats which received neonatal control treatments follow by ABI with zymosan or LPS (the Anesth + LPS.ABI and Anesth + ZY.ABI groups) at the doses/times employed. This information is summarized in Figure 5.

### Section Summary

At the dosing and timing parameters employed in this set of studies, treatment with intravesical zymosan but not intravesical LPS on days P14-16 led to augmented VMRs following a second bladder inflammatory insult as an adult. It is notable that intravesical LPS and intravesical zymosan were both effective as second insults–each producing increases in the vigor of VMRs to UBD when compared with their appropriate control treatments. However, rats treated as neonates with intravesical LPS did not differ from rats given control treatments as neonates (when matched by their adult treatments) whereas rats treated as neonates with intravesical zymosan did differ from their corresponding control rats. This would argue that not only is “bladder inflammation” important but the “type” of bladder inflammation matters, too, in relation to the neonatal insult. The primary caveat of these studies was that an exhaustive study of multiple variables (e.g., concentration of LPS; strain of *E.Coli*) was not performed in the same way as previously done for zymosan (24). A dose/strain of LPS was used that has been reported as effective at producing bladder inflammation by others [e.g., (31)] and in the present study, an ABI effect of LPS treatment in adults was observed suggesting that the regimen utilized was appropriate to the question addressed.

## Question #4: Is ABI the Only Effective Second Insult Following NBI?

### Background

In all of our published studies related to the NBI model, the “second insult” which was employed was a repeat bout of bladder inflammation 24 h prior to testing. This ABI pretreatment was used to evoke robust VMRs to UBD as well as other observations which were noted in the Introduction. However, clinical data related to IC/BPS suggests that multiple different antecedent events experienced in adulthood are correlated with the development of IC/BPS and of “flares” in the magnitude of the symptoms associated with that disorder (pain and/or urinary frequency) [e.g., (32, 33)]. These antecedent events include bladder infections (the rationale for using ABI), acute stress, surgery and other pathological processes including inflammatory and neuropathic pain of non-bladder structures (34). Of these, it is notable that episodes of acute anxiety/stress are the most common “triggering” events apart from recurrent urinary tract infections for flares in symptoms in subjects with IC/BPS (20). To determine whether other non-bladder events could lead to an augmentation of VMRs to UBD in rats which had experienced NBI, we utilized multiple models of pain as second insults: a model of non-bladder inflammatory pain; a model of neuropathic pain; and a model of acute stress/anxiety. Selection of these models was based on their extensive use in our laboratories [e.g., (35, 36)].

### Methods

#### General

All rats in these studies underwent Neonatal Treatments as described in section Neonatal treatments using only zymosan on days P14-16 as an inducer of NBI. As adults, they then went through one of the protocols described below in Sections Acute footshock-induced stress, inflammation of the hindpaw or Chronic constriction injury of the infraorbital nerve and then had VMRs to UBD determined according to the protocols described in section Visceromotor Response (VMR) Measures and statistical analysis as described in section Quantitative analysis.

#### Acute Footshock-Induced Stress

We have characterized stress-induced bladder hyperalgesia in female rats using acute footshock (AFS) as a stressor (35, 37) and the same methodology was used here. This paradigm has been demonstrated to produce increased anxiety-evoked behaviors, activation of the hypothalamic-pituitary-adrenal axis and augmented UBD-evoked VMRs. The present study examined whether NBI altered the vigor of these responses and so all rats in these studies underwent Neonatal Treatments as described in section Neonatal treatments using only zymosan on days P14-16 as an inducer of NBI. As adults (12–15 weeks of age), rats were placed in operant conditioning chambers enclosed in sound-attenuating cubicles for 15 min on six separate adaptation sessions in the days before a final session on the day of testing. On that day, they were divided into two groups: the AFS group was placed in test chambers where they received intermittent footshock (15 min period, 30 random interval footshocks, 1 mA, 1 s duration), the No FootShock (NFS) group were placed in the same chambers but received no footshock. Both groups had VMRs to UBD measured under urethane anesthesia (1.2 gm/kg i.p. with low dose, 0.25% isoflurane but otherwise as per section Visceromotor Response (VMR) Measures) immediately following their final treatment and statistical analysis performed as in section Quantitative analysis.

#### Inflammation of the Hindpaw

The present study examined whether NBI and adult hindpaw inflammation altered the vigor of VMRs to UBD and so all rats in these studies underwent Neonatal Treatments as described in section Neonatal treatments using only zymosan on days P14-16 as an inducer of NBI. As adults (12–15 weeks of age) rats were anesthetized with inhaled isoflurane (2–5%), the skin of their hindpaw prepped with a povidone/iodine solution and 0.1 ml of Complete Freund's Adjuvant (CFA; Sigma-Aldrich, St. Louis, MO) was injected into the dorsolateral hindpaw using a 30 gauge needle. Control injections (VEH) utilized the same methods but injected 0.1 ml of vehicle (mineral oil; Sigma-Aldrich, Inc.; St. Louis, MO) into the hindpaw instead. Rats were allowed to recover from anesthesia and 3 days later had VMRs to UBD measured as described in section Visceromotor Response (VMR) Measures and statistical analysis performed as in section Quantitative analysis.

#### Chronic Constriction Injury of the Infraorbital Nerve

The present study examined whether NBI and chronic facial neuropathic nociception altered the vigor of VMRs to UBD and so all rats in these studies underwent Neonatal Treatments as described in section Neonatal treatments using only zymosan on days P14-16 as an inducer of NBI. As adults (10 weeks of age) rats received bilateral ligation of the Infraorbital Nerve branches of the Trigeminal Nerve utilizing the methodology described elsewhere [e.g., (36, 38, 39)] or underwent a Sham surgery in which incisions were performed and the connective tissue around the nerves was manipulated, but no ligation was performed. Briefly, under inhaled isoflurane anesthesia (2–5%) the scalp skin in the periorbital region had hair clipped and was prepped using a povidone/iodine solution. Incisions/retractors exposed the infraorbital nerves as they traversed the orbital region and 5-0 absorbable chromic suture was used to perform two ligations on each side approximately 5 mm apart. Rats also received a single dose of ampicillin (50 mg/kg s.c. on day of surgery) and three doses of carprofen (5 mg/kg s.c. daily starting on the day of surgery). Rats were allowed to recover from this surgery for 4 weeks as we have previously demonstrated that is the time when they begin to develop signs consistent with facial cold and mechanical hypersensitivity [e.g., (36, 38, 39)]. Others have documented spontaneous signs of facial pain such as grimacing (40) indicating ongoing, spontaneous pain due to Infraorbital Nerve Chronic Compression (-IoNCC). VMRs to UBD were obtained as described in section Visceromotor Response (VMR) Measures and statistical analysis performed as in section Quantitative analysis.

### Results

#### Acute Footshock Stress Results in Augmented VMRs to UBD in Rats Which Experienced NBI

**Figure 6:**
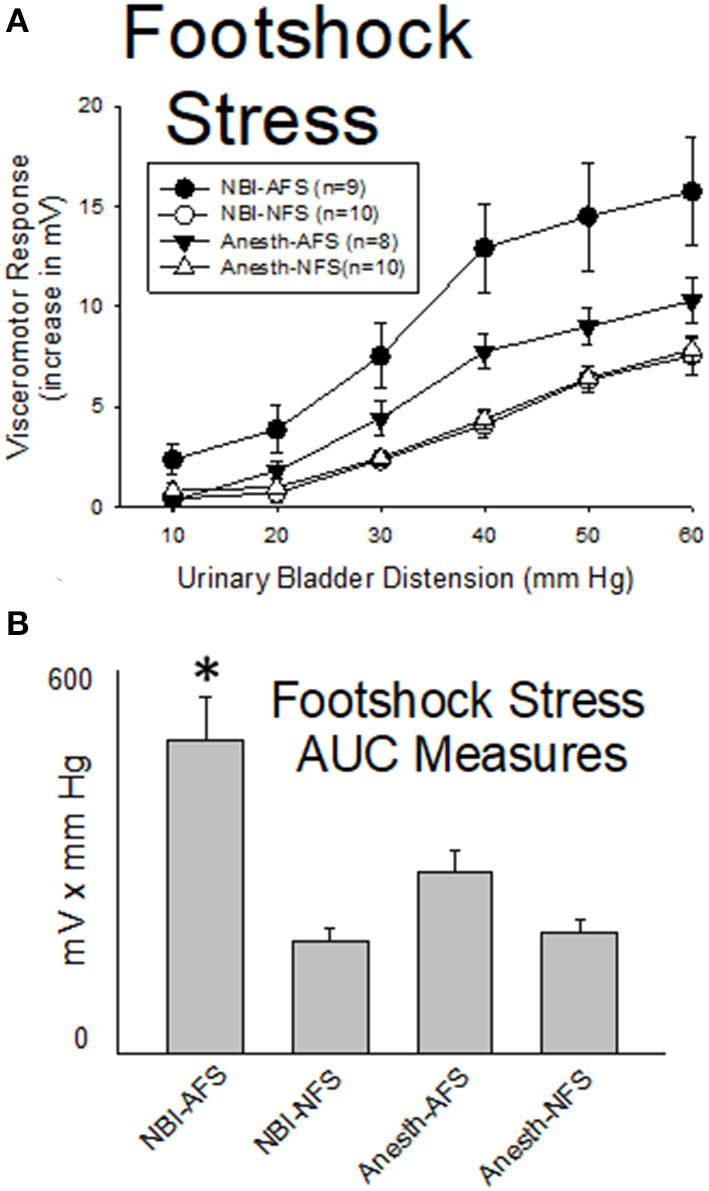
Footshock Stress Augments VMRs in Rats Which Experienced NBI. Presentation is similar to that of Figures 2–4 (group stimulus-response functions in **(A)**; Area-Under-the-Curve, AUC, measures in **(B)**). Rats which had experienced NBI demonstrated statistically greater VMRs to UBD compared with controls when presented with acute footshock stress (AFS) as a second insult as adults. *indicates VMRs in the NBI-AFS group was statistically more robust than all other groups (*p* < 0.05). See text for greater explanation and analysis.

Previous studies have demonstrated that Acute Footshock Stress (AFS) produces augmented VMRs to UBD in adult female rats (35) which were naïve to any additional interventions. As demonstrated in Figure 6, such augmentation was also observed in adult rats which had experienced control treatments as neonates when compared with similarly treated control rats that had No Footshock (NFS) as adults (Anesth-AFS group vs. Anesth-NFS group were different; *p* = 0.014). This augmentation, which was produced by AFS, was magnified further in rats which had experienced NBI. A statistical comparison demonstrated that VMRs to UBD in the NBI-AFS group were more robust than those of the NBI-NFS group (*p* = 0.001), the Anesth-AFS group (*p* = 0.032) and the Anesth-Anesth group (*p* = 0.014).

#### Hindpaw Inflammation Results in Augmented VMRs to UBD in Rats Which Experienced NBI

**Figure 7:**
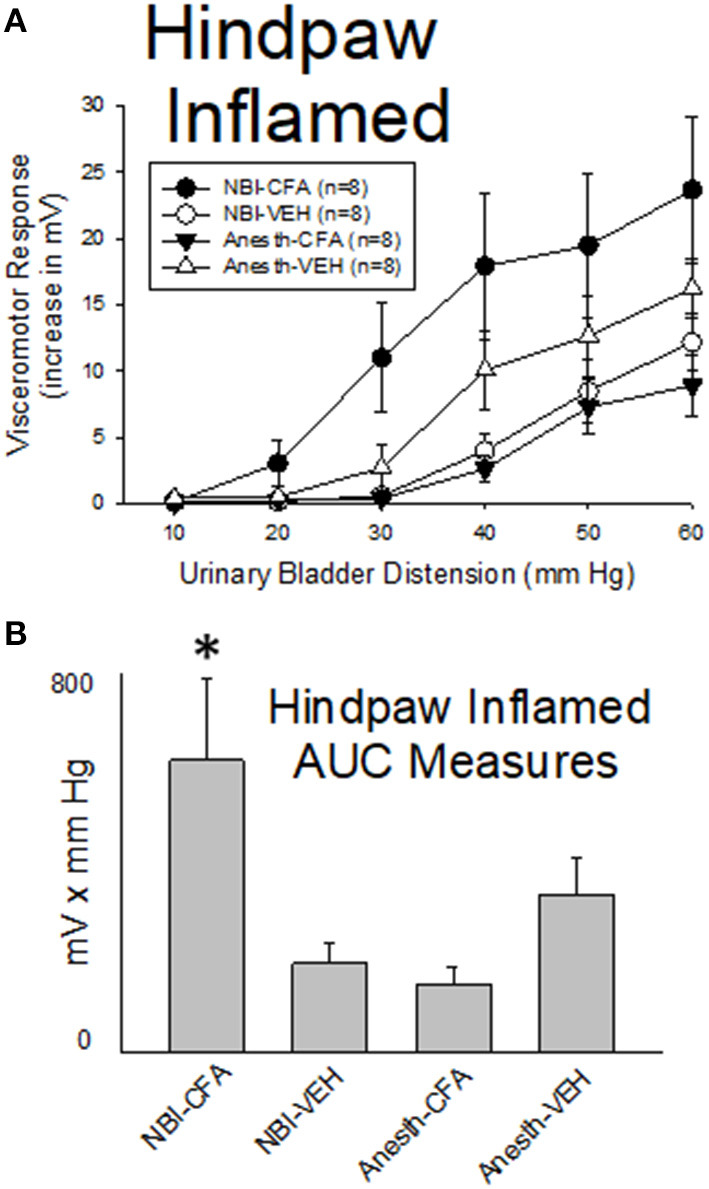
Hindpaw Inflammation Augments VMRs in Rats Which Experienced NBI. Presentation is similar to that of Figures 2–5 (group stimulus-response functions in **(A)**; Area-Under-the-Curve, AUC, measures in **(B)**). Rats which had experienced NBI demonstrated statistically greater VMRs to UBD compared with controls when presented with hindpaw inflammation induced by the injection of Complete Freund's Adjuvant (CFA) as a second insult as adults. “VEH” indicates vehicle injection into the hindpaw as an adult control procedure. *indicates VMRs in the NBI-CFA group was statistically more robust than all other groups (*p* < 0.05). See text for greater explanation and analysis.

As demonstrated in Figure 7, hindpaw inflammation produced by Complete Freund's Adjuvant (CFA; injected 3 days prior to testing) resulted in a *reduction* in the vigor of VMRs in adult rats which had experienced control treatments as neonates when compared with similarly treated neonatal control rats that had vehicle injections as adults (Anesth-CFA group vs. Anesth-VEH group were different; *p* = 0.041). In contrast, CFA injection resulted in an augmentation of VMRs to UBD in rats which had experienced NBI (NBI-CFA group) which was more vigorous than all other tested groups. A statistical comparison demonstrated that VMRs to UBD in the NBI-CFA group were more robust than those of the NBI-VEH group (*p* = 0.001), the Anesth-CFA group (*p* = 0.001) and the Anesth-VEH group (*p* = 0.012).

#### Facial Neuropathic Pain Results in Augmented VMRs to UBD in Rats Which Experienced NBI

**Figure 8:**
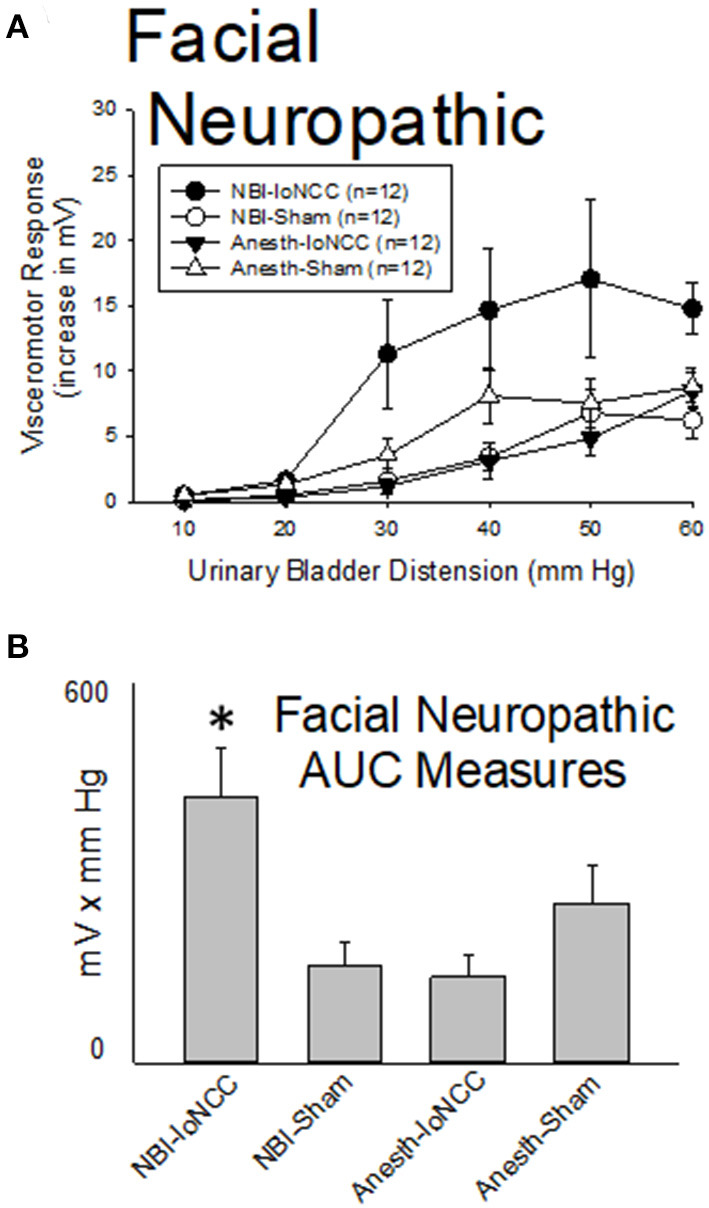
Facial Neuropathic Nociception Augments VMRs in Rats Which Experienced NBI. Presentation is similar to that of Figures 2–6 (group stimulus-response functions in **(A)**; Area-Under-the-Curve, AUC, measures in **(B)**). Rats which had experienced NBI demonstrated statistically greater VMRs to UBD compared with controls when they also experienced InfraOrbital Nerve Chronic Compression (IoNCC) as a second insult as adults. “Sham” indicates sham surgery as an adult control procedure. *indicates VMRs in the NBI-IoNCC group was statistically more robust than the NBI-Sham and Anesth-IoNCC groups (*p* < 0.05). See text for greater explanation and analysis.

As demonstrated in Figure 8, facial neuropathic nociception produced by chronic bilateral ligation of the infraorbital branch of the trigeminal nerve (-IoNCC; 4 weeks prior to testing) resulted in a reduction in the vigor of VMRs in adult rats which had experienced control treatments as neonates that was not statistically significant when compared with similarly treated control rats that had Sham surgeries as adults (Anesth-IoNCC group vs. Anesth-Sham group were not different: *p* = 0.096). In contrast, IoNCC resulted in augmentation of VMRs in rats which had experienced NBI (NBI-IoNCC group) which was more vigorous than the most important control groups. A statistical comparison demonstrated that VMRs to UBD in the NBI-IoNCC group were more robust than those of the NBI-Sham group (*p* = 0.005) and the Anesth-IoNCC group (*p* = 0.002).

### Section Summary

Rats which had experienced NBI demonstrated statistically greater VMRs to UBD compared with controls when presented with a second insult as adults. This was true when the second insult was bladder reinflammation (as noted in previous sections), as well as by anxiety/stress induced by AFS, hindpaw inflammation produced by CFA or facial neuropathic nociception produced by IoNCC.

## Question #5: Does NBI Produce Any Other Features Observed Clinically in IC/BPS?

### Background

Despite the heterogeneous nature of the IC/BPS population, deep phenotyping of individuals have identified numerous features that are more common in subjects with the diagnosis of IC/BPS than in matched control subjects (20, 41). Notably, none are pathognomonic or universal for the disorder but simply more likely to be present. These include increased anxiety/stress measures, increased sensitivity to intravesical chemical stimuli particularly those high in potassium, the presence of vascular fragility in the bladder itself, manifest as glomerulations (submucosal petechial hemorrhages) following hydrodistension of the bladder and increased pelvic floor muscular sensitivity and tone. Using standard methods for assessment we examined whether any “rat-equivalent” signs consistent with human signs/symptoms were present in rats treated with NBI. These methods consisted of the following: (1) an assessment of ongoing stress/anxiety by utilizing an Elevated Plus Maze; (2) an assessment of sensitivity to intravesical potassium solutions using VMRs as endpoints; (3) a histological assessment of bladder tissues following sustained hydrodistension of the bladder; and (4) EMG activity (both baseline and as VMRs to UBD) of the left pubococcygeus muscle, one component of the pelvic floor musculature.

### Methods

#### Elevated Plus Maze

All rats in these studies underwent Neonatal Treatments as described in section Neonatal treatments using only zymosan on days P14-16 as an inducer of NBI. As adults (>12 weeks), all rats were placed on an elevated two arm “plus maze” apparatus with one open arm (50 x 10 cm) and one enclosed arm (50 x 10 x 27 cm) with a junctional area (central area) of 10 x 10 cm, elevated to a height of 52 cm above the floor. To assess anxiety-like behaviors, rats were placed facing the closed arm in the central area and their behavior was video recorded. Percentage of time spent in the open arm was quantified by two separate observers who viewed the video recordings independently and were not aware of group assignment. The average values of their observations were utilized for statistical analyses. Minimal/maximal time in open arm measures was limited to those between 5 and 60 s for statistical analysis. Simple unpaired *t*-tests were used to compare groups.

#### Intravesical Sensitivity to Potassium Chloride

All rats in these studies underwent Neonatal Treatments as described in section Neonatal treatments using only zymosan on days P14-16 as an inducer of NBI. As adults, all rats were deeply anesthetized with inhaled isoflurane (2–5%), a 22 gauge angiocatheter was placed intravesically via the urethra and rats surgically prepared for measures of abdominal EMG activity as described above for VMRs in section Visceromotor Response (VMR) Measures. Isoflurane anesthesia was reduced to approximately 1% until withdrawal reflexes to toe pinch returned. The EMG was monitored continuously for 6 min and then 100 microliters of normal saline was instilled into the bladder and drained after 4 min. Following an additional 1 min measure of EMG activity 100 microliters of 0.4 mM potassium chloride (KCl) solution was instilled into the bladder and then drained after 4 min. EMG activity following instillation of either normal saline or KCl solution was quantified as the change in rectified EMG activity measured in microvolts in 1 min epochs and statistically analyzed in a fashion similar to that described in section Quantitative analysis.

#### Histological Effects of Hydrodistension

One set of rats were used for a “range-finding” portion of this study; these rats were naïve to any previous treatments. However, a separate group of rats used to examine NBI effects underwent Neonatal Treatments as described in section Neonatal treatments using only zymosan on days P14-16 as an inducer of NBI. As adults, all rats were deeply anesthetized with inhaled isoflurane (2–5%), a 22 gauge angiocatheter was placed intravesically through the urethra and secured using a purse string suture around the urethral orifice. The transurethral catheter was attached to a fluid-filled column of warm (37°C) normal saline and the height of the top of the fluid column adjusted to be between 40 and 60 cm above the height of the bladder. Distension of the bladder by this fixed pressure of normal saline was maintained for 30 min after which the fluid was drained. Rats were then kept anesthetized for another 30 min after which they were euthanized by bilateral thoracotomy and cardiac section. Fresh bladders were removed and cut lengthwise and spread out onto a coded microscope slide. A blinded observer then examined the slides and recorded the number of sites of punctate bleeding which were visible in each bladder.

#### Pubococcygeus EMG Activity

All rats in these studies underwent Neonatal Treatments as described in section Neonatal treatments using only zymosan on days P14-16 as an inducer of NBI. Methodology identical to the VMRs described in section Visceromotor Response (VMR) Measures above, was used to generate the EMGs with the exception that the intramuscular wire electrodes used to generate EMGs were placed in the left pubococcygeus muscle rather than the superior oblique musculature and urethane anesthesia (1.2 gm/i.p.) supplemented with low dose isoflurane (typically 0.25%) was used as the anesthetic.

### Results

#### Elevated Plus Maze Measures Indicate Increased Anxiety in Rats Which Experienced NBI

Rats Which Experienced NBI on P14-16 Demonstrated a Reduced Time in the Open arm in Elevated Plus Maze (22.6 ± 1.8 s; *n* = 39) When Compared With Neonatal Anesth- Control Rats (29.9 ± 2.3 s; *n* = 32; Difference Between two Groups *p* = 0.014, Unpaired *t*-Test).

#### Intravesical Potassium Produced Robust VMRs in Rats Which Experienced NBI

**Figure 9:**
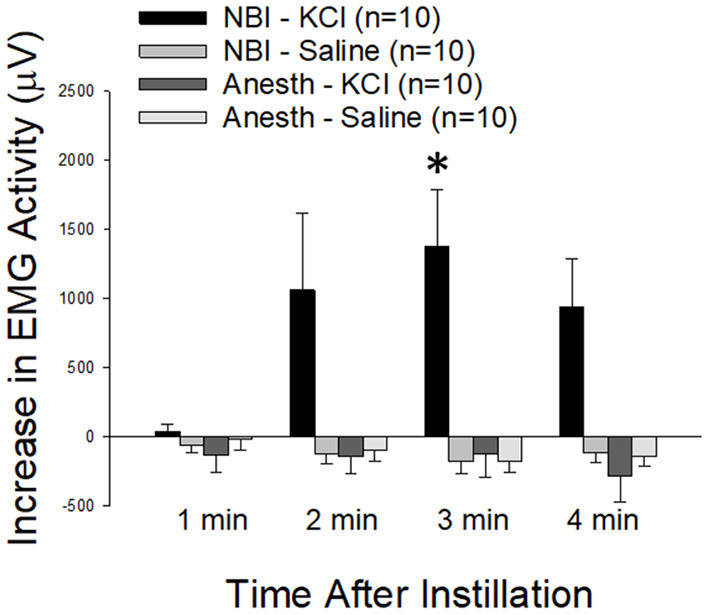
Effect of NBI on Responsiveness to Intravesical Potassium Chloride Solutions. VMRs to the intravesical infusion of 100 microliters of either normal saline (Saline) or potassium chloride (KCl; 0.4 mM) solutions measured as EMG activity averaged over 1 min epochs differed between rats which experienced NBI vs. neonatal control (Anesth) treatments. * indicates statistically significant difference between the NBI-KCl group and all other measures at the 3 min timepoint (*p* < 0.01).

As demonstrated in Figure 9, the intravesical infusion of 100 microliters of a 0.4 mM KCl solution produced a robust VMR in rats which had experienced NBI on days P14-16 (the NBI-KCl group; change from baseline EMG activity was statistically significant at 3 min following infusion; *p* = 0.008, repeated measures ANOVA). Infusion of normal saline did not evoke similar responses in these same animals (NBI-Saline group) and rats which had not experienced NBI had no response to either saline or KCl solution infusions (Anesth-Saline and Anesth-KCl groups). A statistical comparison of change from baseline activity demonstrated that data related to VMRs in the NBI-KCl group were significantly different from all three other groups at the 3 min timepoint (comparison with NBI-Saline group *p* = 0.003, with Anesth-KCl group *p* = 0.001 and with Anesth-Saline group *p* = 0.001). An averaging of the increases in activity during the entire 4 min of fluid infusion demonstrated similar statistically significant differences between the NBI-KCl data (mean 856 ± 252 μV) and the other three sets of data (NBI-Saline data: mean−171 ± 146 μV, difference from NBI-KCL group *p* = 0.002; Anesth-KCl data: mean−121 ± 58 μV, difference from NBI-KCl group *p* = 0.001; Anesth-Saline data: mean−109 ± 70 μV, difference from NBI-KCL group *p* = 0.002).

#### Rats Which Experienced NBI Had Increased Vascular Fragility in the Bladder

Initial studies in rats which had received no pretreatments were used to determine the optimal hydrodistension pressures for generating submucosal petechial hemorrhages in the bladders. In these “naïve” rats (*n* = 9/group): 40 cm H_2_0 pressure hydrodistension produced 0.4 ± 0.2 hemorrhages/bladder; 50 cm H_2_0 hydrodistension produced 1.1 ± 0.3 hemorrhages/bladder; 60 cm H_2_0 hydrodistension produced 4.7 ± 1.1 hemorrhages/bladder; 80 cm H_2_0 hydrodistension produced 9.3 ± 2.2 hemorrhages/bladder. Based on these data, we utilized 50 cm H_2_0 pressure for subsequent experiments considering that it would be the optimal hydrodistension pressure for examining differential effects of NBI on bladder vascular fragility since it was the pressure where “damage” effects were first reliably noted. When 30 min of 50 cm H_2_0 hydrodistension was utilized in adult rats which had experienced NBI, 6.0 ± 1.1 hemorrhages/bladder were observed. This was statistically > the 2.0 ± 0.4 hemorrhages/bladder that were observed with the same treatment in rats which had only received neonatal Anesth- control treatments on P14-16 (difference *p* = 0.0034; unpaired *t*-test; *n* = 9 in both groups).

#### Pelvic Floor Tone and Responsiveness Are Increased in Rats Which Experienced NBI

As reported in Table 2, basal EMG activity (tone), measured as a rectified EMG of the left pubococcygeus muscle prior to any UBDs, was significantly greater (*p* = 0.018 for difference) in rats which experienced NBI on P14-16 (the NBI-ABI and NBI-Anesth groups combined) when compared with rats which only received Anesthesia (the Anesth-ABI and Anesth-Anesth groups combined). The same was true of EMG activity during 20 s of 60 mm Hg UBD (*p* = 0.018 for difference) as well as the Evoked Response (increase over basal level; *p* = 0.027 for difference). With the present sample size, there were no statistically significant differences between the more specific subgroups. It is notable that the EMG activity of the pubococcygeus muscle appeared elevated in the NBI-Anesth group in relation to other groups whereas this group had the same or lower EMG activities than the other groups when the abdominal musculature was being used to measure VMRs.

**Table 2:** Electromyographic activity (mean mV) of the left pubococcygeus muscle in female rats which had experienced NBI and/or ABI.

**Group (*n*)**	**Basal “Tone”**	**During UBD**	**Evoked response**
NBI-ABI(18)	295 ± 67	1,302 ± 223	1,008 ± 194
NBI-Anesth (9)	253 ± 83	1,126 ± 508	874 ± 513
Anesth-ABI (9)	157 ± 48	548 ± 152	391 ± 130
Anesth-Anesth (9)	81 ± 20	528 ± 192	448 ± 188
All NBI Combined (27)	280 ± 50^*^	1,241 ± 219^*^	961 ± 178^*^
All Anesth- Combined (18)	119 ± 27	538 ± 119	419 ± 111

*NBI, Neonatal Bladder Inflammation; ABI, Adult Bladder Inflammation; Anesth- represents control treatments as a neonate. -Anesth indicates control treatments as an adult. Data are presented as mean ± SEM.*

* *indicates significant difference from All Anesth- group (p < 0.05; paired t-test)*.

### Section Summary

NBI experienced on P14-16 was associated with increased measures of anxiety, increased responsiveness to intravesical potassium, increased bladder vascular fragility and increased pelvic floor muscle tone/reactivity to UBD. These are all features that have been noted in humans with the diagnosis of IC/BPS (20).

## Discussion

The most important finding of the present study was that a model system which produced phenotypic features similar to those of the human disorder IC/BPS, was further validated and characterized in a way which argues for its use in the study of mechanisms and treatment of IC/BPS. Consistent with the scientific panel goals delineated by Lai et al. (1), we observed enhanced nociceptive responses to UBD, evidence of enhanced pelvic nociception and increased urinary frequency. An added bonus was that multiple phenotypic features were observed that correlate with those of IC/BPS including an augmentation/flare in responses to UBD produced by stress, nearby inflammation and neuropathic pain inputs. It is notable that many of these features (e.g., increased spontaneous micturition) were present even prior to a second insult such as ABI.

There is epidemiological evidence of a mechanistic link to this model system since it has been reported that childhood bladder inflammatory events such as urinary tract infections and antibiotic use (which may alter mycotic flora) is reported as more frequent in subjects who develop IC/BPS as adults (42–46). The female predominance of IC/BPS is also correlative with childhood bladder infections which are more common in females (29). A limitation of the present studies is that they were performed only in female rats. This limits any extrapolation to male pelvic pain syndromes.

There are numerous other questions that can be raised related to the zymosan-induced NBI double insult model, many of which may need to be answered by rhetorical rather than experimental means. For example, we used a 1% solution of Zymosan A because previous methodological studies had demonstrated lesser evidence of inflammation with lesser concentrations and greater toxicity without greater bladder inflammation at higher concentrations (24). Peak inflammatory effects of the zymosan, measured as Evans Blue extravasation in the bladder, were present 24 h after treatment but returned to normal levels by 48 h. Hence, NBI treatments performed at 24 h intervals would seem both rational and prudent, although a formal parametric analysis was not performed. Whether zymosan is the best bladder inflammatory agent as opposed to other agents is still an open-ended question. As noted above, we did not observe similar effects of a second insult in rats which received neonatal treatments with intravesical LPS rather than zymosan, a result which was surprising to us. This result may be due to the fact that zymosan produces inflammation via activation of TLR2 and TLR6 receptors whereas LPS acts mainly through TLR4 receptors (25) and so the “type” of inflammation may be important in relation to subsequent phenotypic changes. However, our results may also represent a methodological issue with the strain of *E.Coli* used or the concentration of the LPS and/or duration of exposure since these parameters were not parametrically examined. That said, given our current observations, the model system using zymosan will continue to be used in our laboratory because robust and reproducible results were obtained.

UBD pressures of 10–30 mm Hg are considered “non-noxious” since they are within normal physiological ranges. UBD pressures of 40–60 mm Hg are generally considered to be noxious intensities of stimulation. The present study gave evidence that pressures in these higher ranges (50 cm H_2_O = 37 mm Hg in pressure measures), when sustained for 30 min, can produce actual tissue damage as evidenced by the production of submucosal hemorrhages. This would support the Sherringtonian definition of noxious stimuli as those which produce or predict tissue damage (47). In our experiments, a pressure of 50 cm H_2_0 was borderline noxious in normal rats but definitely noxious in rats which had experienced NBI on P14-16 as they had a greater number of hemorrhages (actual tissue damage) evoked by a 50 cm H_2_0 hydrodistension.

Other methodological questions not addressed in the present set of studies included the optimal age for adult study. Notably, we have performed a brief investigation of VMRs measured in 60 day old “adult” rats (rather than 90–105 day old rats) who had been treated with intravesical zymosan on P14-16 and found the VMRs to be less reliable and with less of an effect of the NBI treatment (data not shown) which convinced us to continue using older rats for study. It did make us wonder whether allowing rats to get even older would lead to even more robust results or whether aging could also serve as a second insult. However, examination of that variable will be left to a future study. The results of the present study narrows the previous “window of vulnerability” from <28 days to >9 to <21 days and so reaffirms our current use of the P14-16 window.

**Figure 10:**
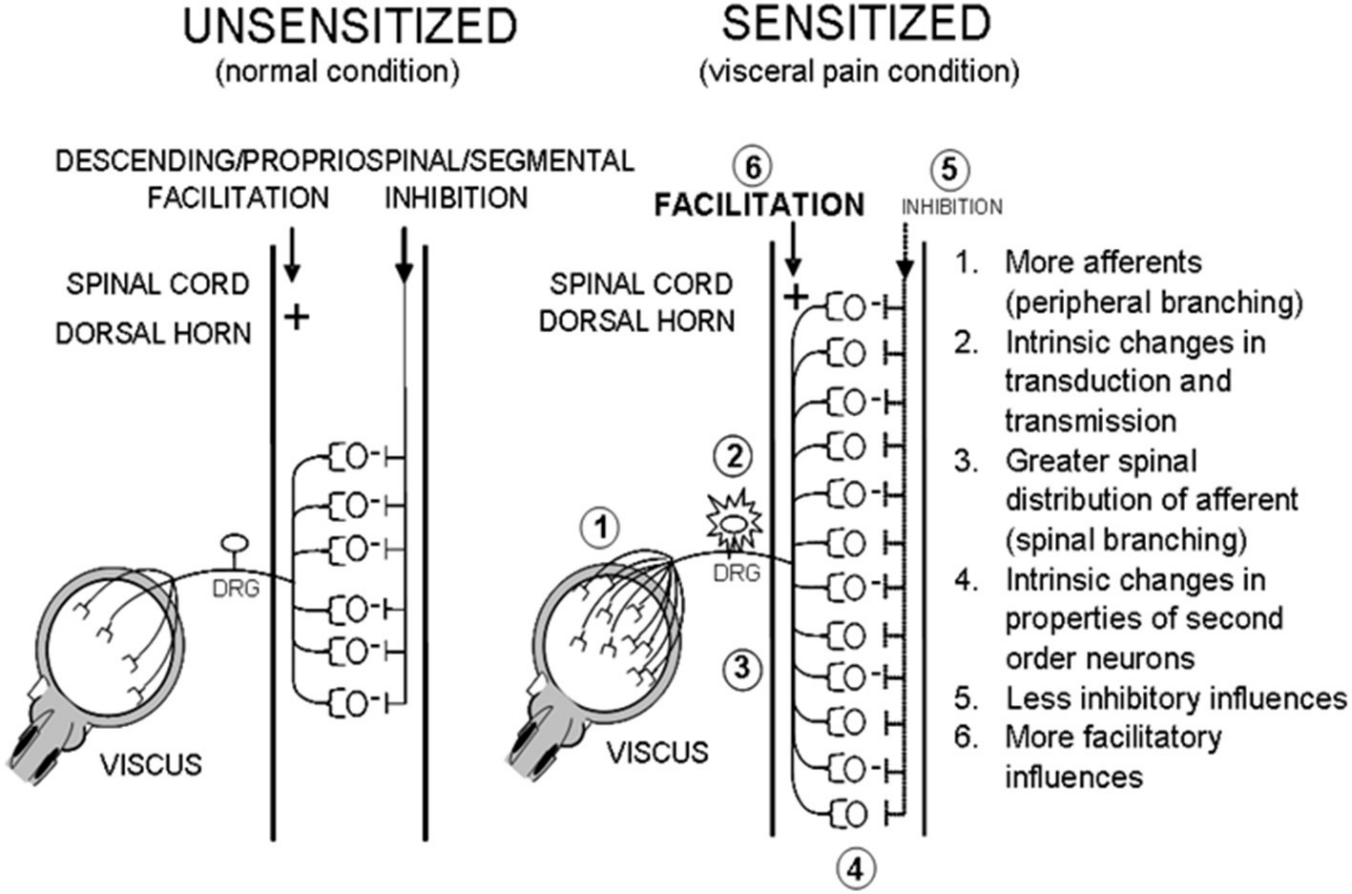
Potential Effects of NBI. Hypothetical mechanisms that lead to a sensitized state that might occur secondary to an episode of NBI.

What happens to the rat following an episode of NBI is still to be fully elucidated. At a spinal level, numerous changes could occur that cause the organism to move into a “sensitized” state such that a second insult as an adult results in increased nociceptive responses to bladder stimuli. Some of these are delineated in Figure 10. To date, we and others have identified that some of these possibilities have an anatomic and/or physiological basis and may involve subsets of bladder afferents (48, 49). For purposes of the present set of studies, it is sufficient to say that various optimal parameters related to NBI treatments have been defined and should be utilized in future studies attempting to delineate the mechanisms of NBI-induced sensitization (50–54).

## Conclusions

The present set of studies have examined some of the parameters and effects of NBI induced by the intravesical mycotic cell wall component zymosan. Effects of NBI on responses to urinary bladder distension were noted in adult female rats such that a sensitized state has been identified. The phenotypic findings observed in this model have correlates to the clinical features of IC/BPS in humans and so support use of this model system to examine mechanisms of and treatments for IC/BPS. Future studies should therefore utilize these methodological findings to optimize their methodology in relation to the effects of neonatal/childhood events and IC/BPS.

## Data Availability Statement

The raw data supporting the conclusions of this article will be made available by the authors, without undue reservation.

## Ethics Statement

The animal study was reviewed and approved by UAB IACUC.

## Author Contributions

All authors have contributed to the intellectual content of this manuscript. TN and AR were involved in the study design, data collection, data analysis, and manuscript generation/revision. CD, JD, MH, BC-M, JG, and JL were all involved in data collection and manuscript generation/revision.

## Funding

These studies were supported by a grant from the United States National Institutes of Health – DK51413.

## Conflict of Interest

The authors declare that the research was conducted in the absence of any commercial or financial relationships that could be construed as a potential conflict of interest.

## Publisher's Note

All claims expressed in this article are solely those of the authors and do not necessarily represent those of their affiliated organizations, or those of the publisher, the editors and the reviewers. Any product that may be evaluated in this article, or claim that may be made by its manufacturer, is not guaranteed or endorsed by the publisher.
